# Meaning in life as a protective factor against suicidal tendencies in Chinese University students

**DOI:** 10.1186/s12888-020-02485-4

**Published:** 2020-02-18

**Authors:** Bob Lew, Ksenia Chistopolskaya, Augustine Osman, Jenny Mei Yiu Huen, Mansor Abu Talib, Angel Nga Man Leung

**Affiliations:** 1 Department of Social Psychology, Putra University of Malaysia, Serdang, Selangor Malaysia; 2 Eramishantsev City Clinical Hospital, Moscow, Russia; 3 Department of Psychology, University of Texas at San Antonio, San Antonio, TX USA; 4 Department of Social Work and Social Administration, The University of Hong Kong, Hong Kong, China; 5 Department of Social Psychology, Faculty of Human Ecology, Putra University of Malaysia, Serdang, Selangor Malaysia; 6 Department of Psychology and Centre for Psychosocial Health, The Education University of Hong Kong, Hong Kong, China

**Keywords:** Meaning in life, Protective factor, FDI-24, Hopelessness, Suicide, China

## Abstract

**Background:**

A substantial increase in rates of suicide worldwide, especially among late adolescents and young adults, has been observed. It is important to identify specific risk and protective factors for suicide-related behaviors among late adolescents and young adults. Identifying specific factors across the masses, not only in the Western, but also in the Asian context, helps researchers develop empirically informed intervention methods for the management of protective and risk factors of suicide.

**Methods:**

In the current study, 2074 students (706 males), filled out the Meaning in Life Questionnaire, with subscales of Search for Meaning (MLQ-S) and Presence of Meaning (MLQ-P); the Future Disposition Inventory-24 (FDI-24), with subscales of Positive Focus (PF), Suicide Orientation (SO), and Negative Focus (NF); and the Beck Hopelessness Scale (BHS). These scales measure protective and risk factors that are linked to suicidal behaviors; while suicidal behaviors were measured by the Suicidal Behaviors Questionnaire-Revised (SBQ-R). Mediation analyses were performed to test the models with both the MLQ-S and MLQ-P as the mediators between a) hopelessness, as measured by BHS and suicidal behaviors; and b) PF, SO, and NF, as measured by FDI-24, and suicidal behaviors.

**Results:**

We found that only MLQ-P mediated the relation between hopelessness and suicidal behaviors; while both MLQ-P and MLQ-S mediated PF, SO, and NF (as measured by FDI-24), and suicidal behaviors, respectively.

**Conclusion:**

Meaning in life, including both the presence of meaning in life and search for meaning, can be good protective factors against suicidal behaviors.

## Background

Suicide is a significant public health problem worldwide. According to the estimation of the World Health Organization, about 800,000 people die by suicide every year [1]. In China, in 1987–1994, suicide was the principal contributor to the observed increases in the mortality rates for young adults, ages 20–29 years [2]. Although the rates of suicide decreased slightly in the years 2002–2011 in China [3], more studies are needed to gain an in-depth understanding of the factors that contribute to death by suicide among late adolescents to young adults. Concurrent with emerging adulthood, university time is a period of relative instability and struggling feelings; young people face difficult questions, and they try to understand who they are and what they are going to do with their lives [4]. Most young adults attempt to find meaningful solutions to their developmental difficulties; for instance, they have to adapt to new stressful experiences, such as being away from home, which implies unique coping challenges [5]. Unfortunately, some young adults, including college students, see death by suicide as a viable solution to these stressful life challenges. Studies with college student populations have reported externalizing and internalizing risk factors that are related to suicide, which include poor parent-student relationships, affective dysregulation, substance abuse, academic difficulties, low social support, and depression [6]. In addition, feelings of hopelessness [7, 8] and anxiety [9], as well as low life purpose and meaning of life [10], are the most common internalizing risk factors for undergraduate students to commit suicide.

### Meaning in life and a search for it

As a psychological construct, meaning in life is a significant factor in an individual and is a protective factor against suicidal dispositions [11–16]. Neurologist and psychiatrist Viktor Frankl argued that finding meaning in life is the primary motivating force for any individual. Frankl suggested that we have the free will to search for meaning in our lives, even when we are facing inevitable suffering. He stated further that “meaning is something to be found rather than to be given, discovered rather than invented” ([17], p., 43). Similarly, Steger and colleagues defined meaning in life as “the sense made of, and significance felt regarding, the nature of one’s being and existence” ([18], p., 81).

Since the 1980s, the meaning in life construct has been shown to be a mediator (or a buffer, suppressor) between depression, self-derogation**,** and suicide ideation among student samples [19]: lack of purpose in life mediated the relations between self-derogation and substance abuse, as well as the relations between depression and suicidal thoughts. Meaning in life also was a partial mediator between gratitude, grit, and suicidal ideation in students [20], partially explaining the buffering effect of these constructs on suicidal thoughts. Meaning in life was also a mediator between reasons for living and suicidal ideation in older adults from a community sample [21], which decreases the likelihood of contemplating suicide. In a more recent study, meaning in life, conceptualized as a sense of coherence, was also found to be a moderator between emotion-oriented coping, avoidance-distraction coping, and suicidal manifestations in students, especially in females [22]. Meaning in life in general, especially its life goals and purposes components, moderated distal (e.g., diagnosis of psychiatric disorder, previous attempts), and proximal risk factors (e.g. hopelessness), in a clinical sample of patients with borderline personality disorder [23]. Based on existing literature, meaning in life acts as either a mediator or a moderator. It either explains the influence of factors on suicidal ideation, such as mediating the relations between specific risk or protective factors and suicidal manifestations, or it moderates (by weakening) the relations between risk factors and suicidal thoughts and behaviors. The current study, therefore, included meaning in life as a construct to suppress suicide.

To date, the most widely used self-report instrument to measure the meaning of life is the Meaning in Life Questionnaire (MLQ). It is composed of two subscales: a) Search for Meaning (MLQ-S); and b) Presence of Meaning (MLQ-P) [18]. While the construct of presence of meaning refers to the actual experience of meaning in life, the construct of search for meaning captures the process of looking for and acquiring the meaning in life. The presence of meaning is rather uniformly consented to be beneficial to subjective well-being, but findings on search for meaning are mixed. Steger named search for meaning “a unique and underappreciated dimension of human personality, distinct from more broadband measures of personality and cognitive style, marked by a thoughtful openness to ideas about life” [24]. When individuals need to search for meaning, it seems to imply that there is a lack of meaning in their lives.

Conceptual issue was involved with interpreting scores on the MLQ. A past study has shown that the MLQ-S score was significantly and positively correlated with scores of measures on neuroticism, depression, and negative affectivity, and that it did not correlate with the life satisfaction scale scores, but that MLQ-P scale score was moderately, and positively associated with the life satisfaction scale scores [18]. However, other recent studies found that while the presence of meaning was positively related to life satisfaction, this relation was stronger among those who were high in search for meaning, than those who were low in it [25], which implies the positive role played by the search for meaning as well. A developmental study also showed that search for purpose in life was associated with increased life satisfaction during adolescence and emerging adulthood [26]; and another experimental study on adjustment to stress found that the score of search for meaning was increased in the experimental condition where subjects were asked to think on anticipated stressors in their lives [27]. The authors suggested that meaning serves a buffering function, leaving people in control of their lives and thus making them more stress-resilient. A quest for meaning in life in the face of future stressors helps them understand the upcoming adverse events in more coherent way.

In the Western context, search for meaning in life tends to be different from presence of meaning and show negative or no correlation between the constructs, however, other studies in the Oriental countries suggested positive correlation between the two [28]. We believe that this may be explained by the cultural differences of the Western versus Oriental countries in terms of their predominant analytical versus dialectical thinking.

These differences warrant further investigation in light of studies suggesting that suicide behaviors in China are becoming more similar to countries in the West, notably the United States. Prior studies highlighted a higher female-to-male suicide ratio in China [29, 30] and that Chinese adolescents and young adults were more likely to end their lifelives by suicide than older adults [30]. Perhaps due to recent social and economic developments, a recent study reported that these differences are narrowing as well [3].

Given the likely role played by different cultures, and that the trend in suicidal behaviour in China is similar but different to the Western context, we decided to explore the role played by both search for meaning in life and presence of meaning against suicidal tendencies of Chinese emerging adults.

### Hopelessness and future orientation

Besides meaning in life, another predictor of suicidal behaviors is hopelessness. Snyder et al. ([31], p., 571) defined hope as “a cognitive set that is based on a reciprocally-derived sense of successful agency (goal-directed determination) and pathways (planning to meet goals).”Within this theoretical framework, Snyder’s hope measures could not be used to assess the negative expectations for future life events. On the contrary, hopelessness is generally conceptualized as “negative attitudes or expectations about future life events” ([32], p., 411). It is viewed as a strong predictor of suicidal ideation and behavior [33], which confers risk for suicide-related behaviors [34]. Similarly, the hopelessness measures could not be used to assess the positive attitude of expectation about future life events. Despite questions that continue to be raised about the dimensionality of the most commonly used instrument to measure hopelessness, the Beck Hopelessness Scale (BHS), this widely-used instrument was used in the present study, as hopelessness has been found to be a critical risk factor in predicting suicide among students [32]. This study, therefore, fills the gaps regarding these constructs by using a newer inventory, the Future Disposition Inventory-24 (FDI-24) [35], which evaluates both protective (positive) and negative future-related thoughts and feelings (i.e., dispositions): Positive Focus (PF), Suicide Orientation (SO), and Negative Focus (NF).

The positive dimension of this new instrument focuses on protective responses: optimism, plans for the future, satisfaction with life, and determination in handling problematic situations. The negative dimension evaluates risk responses such as worry, cognitive rigidity, and life dissatisfaction. The suicide orientation dimension evaluates suicide rumination, suicide ideation, and the wish to die. To date, only a few existing studies (e.g., [35]) have employed the FDI-24 in Chinese samples. Indeed, it is important to assess dispositions about future life events, which include both positive and negative orientation, to understand suicidal behaviors [32].

### Aims and hypotheses of the present study

Positive or negative thinking about the future is measured by hopelessness and future dispositions, and it is hypothesized to be related to suicidal behaviors. Finally, the roles of the MLQ-P and MLQ-S in suicidal behaviors have rarely been examined in the existing literature. From the limited past studies, it seems that compared with MLQ-S, MLQ-P was more strongly and positively correlated with positive well-being [25]. The aim of this study, therefore, was to investigate the link between these two scale scores. Thus, three hypotheses for the current study were proposed as follows:

Hypothesis 1: Presence of meaning in life will be positively correlated with search for meaning in life, consistent with the previous Oriental studies of meaning in life, with search for meaning in life correlating with presence of meaning in life, hopelessness measures and suicidal behaviors.

Hypothesis 2: Meaning in life (both presence and search for it, as measured by MLQ-P and MLQ-S) would mediate the relationship between a) hopelessness, as measured by BHS and suicidal behaviors; and b) PF, SO, and NF, as measured by FDI-24, and suicidal behaviors (as measured by SBQ-R).

Hypothesis 3: The mediating effect of MLQ-P would be stronger than that of MLQ-S.

## Methods

### Sample and procedure

The sampling and procedure we used have been reported in previously published studies [35, 36]. We adopted a multi-stage stratified sampling procedure to recruit participants. Students from two public medical universities in Jinan city, Shandong Province, eastern China were recruited, using convenient sampling. The procedure was as follows: first, two faculty colleges with similar background from each university was selected as the primary sampling unit, then, differentiated and separated by grade, from each grade, three or four classes were randomly selected, which became the secondary sampling units. The in-class paper and pencil survey collection was supervised by professionals in questionnaire administration. Participation in the study was voluntary. Participants filled out their basic demographics (age and gender), followed by completing the Chinese versions of instruments listed below in the Instruments section. Information was given on the questionnaires for students to go for referral, if they had emotional disturbances after filling out the questionnaires. No referrals were observed after the study. A total of 2197 self-report questionnaires were distributed, among them, 2074 questionnsired were completed without any missing item on any of the measured variables in this paper.

### Instruments

#### The future disposition inventory (FDI-24)

FDI-24 [35] is a 24-item self-report measure with three 8-item subscales. The items are rated on a 5-point scale (1= “not at all true of me,” to 5= “extremely true for me”). Sample items are, “I expect things to turn out better for me in life” – Positive Focus subscale; “I sometimes think that by ending my life, all the problems ahead of me will go away” – Suicide Orientation subscale; and “I worry that things will never go well for me no matter what I do” – Negative Focus subscale. The questionnaire was translated and adapted for use with Chinese samples and had adequate indexes of fit for the current sample (R-CFI = .945, R-TLI = .939, R-RMSEA = .096) [35]. Also, the Cronbach’s alpha estimates for the Positive Focus Scale (.916), the Suicide Orientation Scale (.933), and the Negative Focus Scale (.850) for the study samples were acceptable for researched-related analyses.

#### The Beck hopelessness scale (BHS)

The BHS is a 20-item self-report instrument that is designed to assess negative attitudes about future events [37]. The instrument has established estimates of test-retest reliability and construct validity in Chinese samples [37]. In brief, the BHS includes nine positively-worded items and 11 negatively-worded items concerning negative attitude about the future. The total score is derived to evaluate levels of the hopelessness construct; higher total scores represent extreme levels of hopelessness. Used as a criterion-related validation instrument in the current study, the estimate of internal consistency of the BHS score for the study sample was adequate (Cronbach’s alpha = .898). Based on the original scale, an earlier study modified the response format of the BHS [38] from yes/no to a 5-point Likert-type scale ranging from 1 (*strongly agree*) to 5 (*strongly disagree*). This form of response scaling is more suitable for Chinese respondents. Scores on the Chinese version of the BHS have satisfactory reliability and validity estimates in adolescent samples [37].

#### Suicidal behaviors questionnaire-revised (SBQ-R [39];)

This scale was used in the present study as a measure of suicidality, encompassing suicide-related thoughts and behaviors. The scale is unidimensional and made up of four items, assessing suicidal ideation and attempts in a lifetime (Item 1), the frequency of suicidal ideation over the past 12 months (Item 2), the threat of suicide attempt (Item 3), and future likelihood of suicidal behavior (Item 4). A sample item is, “How likely is it that you will attempt suicide in the future?” A total score for this measure, ranging from 3 to 18, is obtained by summing the scores of all the items, with higher scores indicating higher levels of suicidality [40]. The cut-off score between suicidal and non-suicidal for the undergraduate sample is identified as a score of 7 (i.e., a score of 7 or above will be classified as suicidal). In this study, the number of people whose cut-off score was equal to or higher than 7 was 31% (*M* = 5.86, *SD* = 2.47, Med = 5), which we assessed as a considerable number of students, who have reported suicidal ideation or behavior. As reported by Osman et al., the reliability estimate of the SBQ-R for the undergraduate sample was reasonable (Cronbach’s alpha = .76). In this study, the Cronbach alpha value was .67.

#### The meaning in life questionnaire (MLQ)

was used to measure attitudes and satisfaction toward life. It is made up of two 5-item subscales [18]: the presence of meaning in life (MLQ-P, “I understand my life’s meaning”) and a search for meaning in life (MLQ-S, “I am seeking for a purpose or mission for my life”). All 10 items are rated from 1 (*absolutely untrue*) to 7 (*absolutely true*). The item scores are summed, with higher scores indicating a higher degree of a presence or a search for meaning in life. The Meaning in Life Questionnaire was adapted on a Chinese sample with satisfactory indices of fit of a two-factor model: χ^2^ (34) = 78.5, *p* < .01; RMSEA = .077, CFI = .96, IFI = .96 [41]. In this study, the Cronbach’s alpha value was .85 for each subscale, showing satisfactory internal consistency. A bi-factor model^1^ specifying a general factor (meaning in life) and two specific factors, presence of meaning in life and search for meaning in life, fitted well to our data, χ^2^ (25) = 260.82, *p* < .01; RMSEA = .067 (95% C.I., .060–.075), CFI = .96, TLI = .93.

### Analysis strategy

Descriptive statistics and bivariate correlations were computed to determine the relations among scores on the measured|study| variables included in this study. Mediation analyses were conducted using PROCESS software [42] to assess if MLQ subscale scores (mediators) mediated the relationship between BHS score / FDI subscale scores (independent variables; IV) and SBQ-R scores (dependent variable; DV). The direct effect (from each independent variable to the dependent variable) and indirect effect (from each independent variable to each mediator and from each mediator to the dependent variable) of eight mediation models were tested, with age and gender controlled. Kappa squared (k^2^) was used to estimate the effect size of the mediation models [43]. For a full mediation model to be supported, the indirect effect must be significant, while the direct effect is no longer significant in the presence of the mediator. A partial mediation model is supported when the indirect effect is significant, where, at the same time the direct effect is still significant in the presence of the mediator.

## Results

The mean age of the participants was 19.79 (*SD* = 1.39), 706 (34%) were males.

Descriptive statistics and correlations among the variables are presented in Table 1. All bivariate correlations were statistically significant (*p* < .001). Hopelessness, Suicide Orientation (SO), and Negative Focus (NF) were positively correlated with suicidal behaviors, while Positive Focus (PF) was negatively correlated with suicidal behaviors. Both the Presence of Meaning (MLQ-P) and Search for Meaning (MLQ-S) were negatively correlated with suicidal behaviors, BHS, SO, NF, and positively connected with PF.

**Table 1:** Correlation matrix, means, and standard deviations of study measures (*N* = 2074)

	1	2	3	4	5	6	7
1. Suicidal Behaviors Questionnaire-Revised (SBQ-R)	–						
2. Positive Focus (PF)	−.18	–					
3. Suicide Orientation (SO)	.28	−.47	–				
4. Negative Focus (NF)	.31	−.30	.70	–			
5. Beck Hopelessness Scale (BHS)	.31	−.53	.40	.49	–		
6. Presence of Meaning (MLQ-P)	−.23	.41	−.28	−.35	−.48	–	
7. Search for Meaning (MLQ-S)	−.13	.38	−.19	−.17	−.33	.60	–
Mean	5.86	30.96	12.17	16.48	46.52	25.95	27.09
SD	2.47	7.26	7.00	6.30	10.00	5.71	5.79

*Note:* All correlations are statistically significant at *p* < .001

**Fig. 1:**
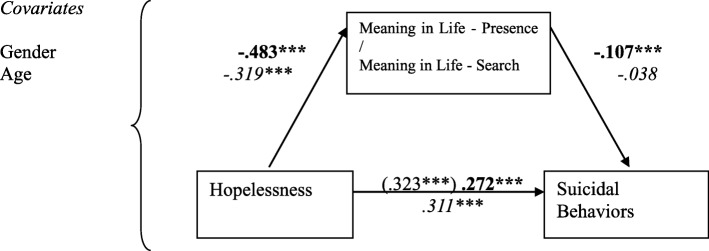
Models with standardized regression coefficients depicting Meaning in Life Presence and Search as mediators in the relation between hopelessness and suicidal behaviors, *N* = 2074. Numbers in bold indicate model with MLQ-P (k^2^ = .048), in *italics* – model with MLQ-S (no mediation). ***p < .001

**Fig. 2:**
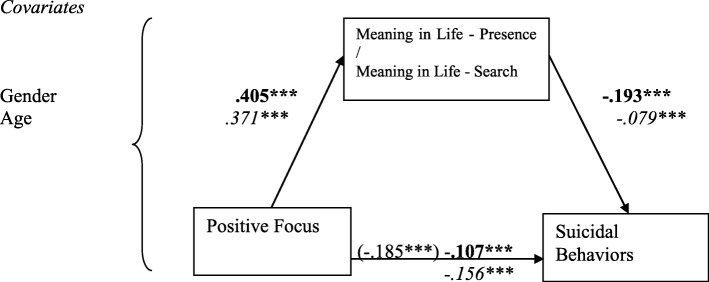
Models with standardized regression coefficients depicting Meaning in Life Presence and Search as mediators in the relation between FDI Positive Focus and suicidal behaviors, *N* = 2074. Numbers in bold indicate model with MLQ-P (k^2^ = .072), in *italics* – model with MLQ-S (k^2^ = .027). ***p < .001

**Fig. 3:**
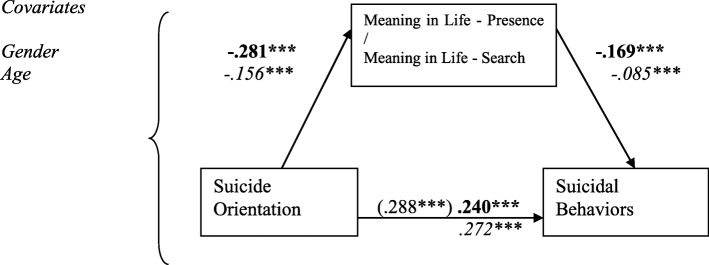
Models with standardized regression coefficients depicting Meaning in Life Presence and Search as mediators in the relation between FDI Suicide Orientation and suicidal behaviors, *N* = 2074. Numbers in bold indicate model with MLQ-P (k^2^ = .048), in *italics* – model with MLQ-S (k^2^ = .016). ***p < .001

**Fig. 4:**
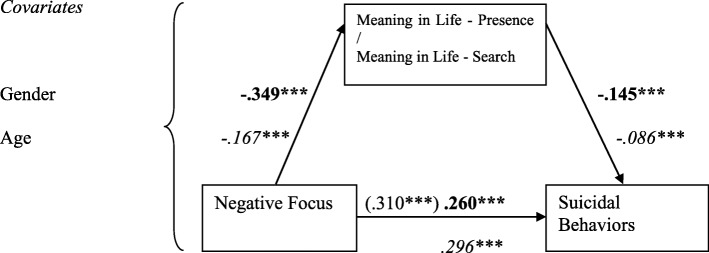
Models with standardized regression coefficients depicting Meaning in Life Presence and Search as mediators in the relation between FDI Negative Focus and suicidal behaviors, *N* = 2074. Numbers in bold indicate model with MLQ-P (k^2^ = .068), in *italics* – model with MLQ-S (k^2^ = .015). ***p < .001

The three FDI subscales, Suicide Orientation (SO), Negative Focus (NF), Positive Focus (PF), and BHS scores (as a measurement of hopelessness) were entered as IVs, with the two MLQ subscale scores (MLQ-P & MLQ-S) as mediators, on suicidal behaviors (measured by SBQ-R, as DV), with gender and age as covariates, yielded eight mediation models. Of them, only seven valid mediation models were found, as the mediation of MLQ-S between BHS and SBQ-R was non-significant (See Figs. 1, 2, 3 and 4).

For MLQ-P, the most salient effect was shown in the mediation model of MLQ-P between PF and SBR-Q, with the effect size equaled .072, while the mediation of MLQ-P between BHS, and SBQ-R, and the mediation of MLQ-P between SO and SBQ-R, both had effect sizes of .048, and the effect size of MLQ-P between NF and SBQ-R = .068. Concerning MLQ-S, the highest mediation effect was also found in the mediation model of MLQ-S, between PF and SBQ-R, with the effect size of.027; while the effect size of the mediation of MLQ-S between SO and SBQ-R = 0.16; and the effect size of the mediation of MLQ-S between NF and SBQ-R = .015.

As we can see, after controlling for gender and age, there were significant negative links between Hopelessness and MLQ-P, and MLQ-P and SBQ-R. The indirect effect for the model with MLQ-P equaled to .051 [.028; .077]. The presence of the meaning in life suppressed the relation between hopelessness on suicidal behaviors. However, there was no mediation found between MLQ-S and suicidal behaviors (Fig. 1).

Controlling for gender and age, we found statistically significant positive connections between PF of the FDI-24 and MLQ-P and MLQ-S scores, and negative links between the meaning subscales and SBQ-R scores. The mediation of Meaning in Life – Presence yielded significant indirect effect −.078 [−.100; −.057], mediation of Meaning in Life – Search had significant indirect effect −.029 [−.009; −.011]. Thus, both Presence of Meaning and Search for Meaning partially explained the negative influence of Positive Focus scores on SBQ-R scores (Fig. 2).

Taking into account gender and age, Suicide Orientation FDI-24 significantly predicted both Meanings in Life – Presence and Search scores, which in turn negatively predicted SBQ-R. Indirect effect for the presence of meaning mediation was .048 [.007; .061], for the search of meaning it was .016 [.005; .025]. Thus, there is an indication that both Presence and Search for Meaning suppressed the influence of Suicide Orientation on suicidal behaviors (Fig. 3).

Taking into account gender and age, Negative Focus subscale of FDI-24 negatively inferred both Meanings in Life – Presence and Search, which in turn negatively predicted SBQ-R scores. The indirect effect of MLQ-P was .050 [.009; .067], of MLQ-S was .014 [.006; .022]; that is, both meaning subscales suppressed the effect of Negative Focus on suicidal behaviors (Fig. 4).

## Discussion

The current study generally supported the three hypotheses. Firstly, the search for meaning in life (MLQ-S) was positively related to the presence of meaning in life (MLQ-P), positive focus, and negatively associated with hopelessness, negative focus, suicide orientation, and suicidal behavior. In this study, an active search for meaning was found to be strongly correlated with the presence of meaning, which is consistent with the findings of other studies in China and Japan [28, 41].

Secondly, as expected and in coherence with the literature, presence of meaning in life acted as a construct to mediate the relations between hopelessness and suicidal behaviors; meaning in life (both presence and search) also mediated between future dispositions (in terms of positive focus, negative focus, and suicide orientation) and suicidal behaviors.

Also, the present study found that with the same set of IVs and DVs, the effect sizes of the mediating models on MLQ-P were a lot stronger than those on MLQ-S (and that MLQ-S did not mediate between hopelessness and suicidal behaviors); therefore hypothesis 3 was supported.

Although MLQ-S did not mediate the relation between hopelessness and suicidal behaviors, MLQ-S mediated relations between all three FDI-24 subscale scores with suicidal behaviors, though to a lesser degree, as compared with the Meaning in Life - Presence (MLQ-P) score. This suggested that both MLQ-P and MLQ-S are important mediators, while the search for meaning can be viewed as a positive, but not a negative factor while assessing suicidality of individuals, and this pattern applied to a non-clinical Chinese population, therefore, hypothesis 3 was mostly supported.

Search for meaning in life does not only act as a schema that increases the salience of meaning-relevant information [25], the current study provided evidence that it also suppressed the negative disposition (Negative Focus and Suicide Orientation). This finding helps clinical consultants draw on this “will to meaning,” as a supportive mechanism for suicidal individuals to form new perspectives to look at their lives from different perspectives. Frankl used this will in his patients to help them understand that if they see no meaning in their lives but crave for it, then it already exists [17]. Taken together, we may go a bit further and see the correspondence to it in the notion of “absent but implicit” in narrative therapy: something that is felt as not there, but still implied as existent, possible [44], attainable in the future.

There have been a number of therapeutic strategies which have explored the meaning in life with clients. For example, the solution-focused therapy technique known as the “miracle question”: the suicidal person is asked to fantasize about the disappearance of their problems [45]; and the Meaning-making processes which are being adopted in constructivist meaning-oriented approach for bereaved individuals [46]. Concerning cognitive behavioral therapy, where the concept of hopelessness was coined, as well as some of its derivatives (as acceptance and commitment therapy), the increase in life meaning is sometimes also being assessed as a desired outcome of the interventions, together with life satisfaction, well-being and quality of life [47].

Most of the aforementioned interventions were proposed by the Western psychotherapies, indeed, some of them were Orientally informed. For example, the dialectical behavioural therapy which helps suicidal people, contains the word “dialectics”, which proposes a “wise mind” (compare to the “Middle Way”) to combat frustrations, to be an effort- and process-oriented, but not being success-driven [48]. Steger claimed that this is indeed what his scales are about: search for meaning is about paying effort, and openness toward new situations, while presence of meaning is about something that a person already has, about stability and perhaps dogmatism [28]. Therefore, linking the two scales could be a promising direction for future studies concerning suicidal behaviour and mental well-being: while one hold what is dear to them (i.e. presence of meaning), one should also be flexible search for meaning in life to face the rapidly changing world, and both will be good protective factors that are against frustrations and feelings of hopelessness [27].

Concerning the psychometric properties of the scales used in this study, the MLQ allows a combination of scoring and interpretations of the two scales. For example, respondents may score high on the MLQ-P but low on the MLQ-S, and vice versa. In our sample, most of the participants scored highly on both scales, and thus the scores on the MLQ could also be interpreted as meaning in life as a whole.

Moreover, the larger effect sizes of mediation of meaning in life variables between the Positive Focus subscale of the FDI-24 and the Suicidal Behaviors Questionnaire-Revised than between the Beck d Hopelessness Scale and the SBQ-R suggested that this subscale is a valuable addition to the measurement of the hopelessness construct. Positive future disposition is not just a reverse of negative future disposition, it has its specific qualitative difference [49], and it does imply a stronger meaning in life and search for meaning, which should be taken into account in screening procedures.

The negative subscales scores of the FDI-24 are a bit more sensitive in the sense that they capture the Search for Meaning, which mediates their relationships with suicidal behaviors, while the Beck Hopelessness Scale does not. This finding indirectly suggests that the FDI-24 may be a more sensitive instrument to use in future screenings, at least with a Chinese population, in assessing the hopelessness construct and its relationship with meaning in life, than the BHS. Alternatively, the mediation effect of meaning in life, which spans across two different instruments, proves to be a stable and robust phenomenon.

### Limitations and future directions

This study employed a cross-sectional design, which hinders conclusion from cause-and-effect relation to be drawn. Nevertheless, the results of the current study provide guidance for future investigations on any causal relations among the measured variables. Another limitation of the current study is about the age of the participants (*M* = 19.79), with most of them belong to the younger period of emerging adulthood (18–25 years old). As participants share similar age and study conditions, the generalizability of applying these results to the older period of emerging adulthood is limited.

While only age and gender were collected as demographic data and included as covariates in this study, future studies can investigate the role of other viable variables (such as socioeconomic status, life events, medical and/or psychiatric history, family ties, social relationship, living alone or with family or roommates, and so on) which may influence meaning in life as a protective factor against suicidal tendencies in university students.

Nevertheless, relative to other past studies that have investigated the protective roles of suicide, the sample size of the current study is commendable. Aside from corroborating with research demonstrating meaning in life as a resiliency factor against suicide [50], the present findings also contribute to our understanding on the mechanism, on how hopelessness influences suicidal behaviors via meaning in life. While meaning in life has been identified as working closely with hope to influence suicidal ideation [51], findings from this study provide implications for future studies to further investigate such mechanism beyond Chinese population. Using different samples is important, because the dimensions of the meaning in life construct and the link between the two MLQ scale scores may differ as the sample changes. Finally, hope and hopelessness are distinct but correlated constructs [52]. In a Hong Kong Chinese sample, the effect of hopelessness on suicidal ideation was lower in individuals with higher hope and higher in individuals with lower hope [52]. Therefore, interventions to reduce the risk of suicide and suicidal ideation could emphasize reducing hopelessness, and perhaps on strengthening hope via focusing on meaning in life.

## Conclusion

To conclude, this study found that the search for meaning in life (MLQ-S) was positively related to the presence of meaning in life (MLQ-P) and positive focus, and negatively associated with hopelessness, negative focus, suicide orientation, and suicidal behaviors. Second, the MLQ-P mediated the relations between hopelessness and suicidal behaviors; while both the MLQ-P and the MLQ-S mediated between future dispositions (in terms of positive focus, negative focus, and suicide orientation) and suicidal behaviors. The results suggested that meaning in life, including both the presence of meaning in life and search for meaning in life, can serve as good protective factors against suicidal behaviors.

## Data Availability

The datasets used and/or analyzed during the current study are available on reasonable request.

## Abbreviations

BHS: Beck Hopelessness Scale
FDI-24: Future Disposition Inventory-24
MLQ: Meaning in Life Questionnaire
MLQ-P: Meaning in Life Questionnaire, Presence of Meaning subscale
MLQ-S: Meaning in Life Questionnaire, Search for Meaning subscale
NF: Negative Focus
PF: Positive Focus
SBQ-R: Suicide Behaviors Questionnaire-Revised
SO: Suicide Orientation

## Footnotes

1 The bi-factor model provided support for the high factor intercorrelations often being observed between scores for the two factor scales found in Oriental studies including the present study. As per bi-factor modeling, MLQ-P and MLQ-S scale scores as well as total scores could be used in interpreting scores on the MLQ.
